# In the national epidemiological bulletins – a selection from recent issues

**DOI:** 10.2807/1560-7917.ES.2018.23.50.1812131

**Published:** 2018-12-13

**Authors:** Eurosurveillance editorial team

**Affiliations:** 1 European Centre for Disease Prevention and Control (ECDC), Stockholm, Sweden

**Keywords:** infectious diseases, public health, Europe

**Healthcare-associated infections and antibiotic resistance**

Laboratory surveillance of pyogenic and non-pyogenic streptococcal bacteraemia in England, Wales and Northern Ireland: 2017

Health Protection Report; 12(41)

16 October 2018, United Kingdom

https://assets.publishing.service.gov.uk/government/uploads/system/uploads/attachment_data/file/756869/hpr4118_pnp-strptccc.pdf

 

**Food- and waterborne diseases**

Suspected and laboratory confirmed reported norovirus outbreaks in hospitals (England) and norovirus laboratory reports (England and Wales): weeks 44 to 47, 2018

Health Protection Report; 12(44)

7 December 2018, United Kingdom

https://assets.publishing.service.gov.uk/government/uploads/system/uploads/attachment_data/file/762759/hpr4418_noro.pdf

 

Gastroenteritis outbreak in Youth camp in July 2016

Vlaams infectieziektebulletin 2018(1)

November 2018, Belgium

https://www.zorg-en-gezondheid.be/sites/default/files/atoms/files/VIB%202018-1_DEF.pdf

 

**Hepatitides**

 

Laboratory reports of hepatitis A and C in England and Wales, April to June 2018

Health Protection Report; 12(39)

2 November 2018, United Kingdom

https://assets.publishing.service.gov.uk/government/uploads/system/uploads/attachment_data/file/753545/hpr3918_hepAC.pdf

 

**Vaccine-preventable diseases**

 

Tetanus in France between 2012 and 2017

Bulletin épidémiologique hebdomadaire, 42/2018

11 December 2018, France

http://invs.santepubliquefrance.fr/beh/2018/42/pdf/2018_42_1.pdf

 

Elimination of endemic measles transmission in Ireland – but low MMR uptake poses threat

Epi-Insight 2018;19(12)

December 2018, Ireland

http://ndsc.newsweaver.ie/epiinsight/2qqve6p2uaf10gkzp9yxn5?a=1&p=54259404&t=17517774

 

Laboratory-confirmed cases of measles, rubella and mumps, England: July to September 2018

Health Protection Report; 12(42)

23 November 2018, United Kingdom

https://assets.publishing.service.gov.uk/government/uploads/system/uploads/attachment_data/file/758965/hpr4218_mmr.pdf

 

Invasive meningococcal disease in England: annual laboratory confirmed reports for epidemiological year 2017 to 2018

Health Protection Report; 12(38)

26 October 2018, United Kingdom

https://assets.publishing.service.gov.uk/government/uploads/system/uploads/attachment_data/file/751821/hpr3818_IMD.pdf

 

Community outbreak of serogroup B invasive meningococcal disease in Beaujolais (Rhône, France) 2016

Bulletin épidémiologique hebdomadaire, 30-31/2018

9 October 2018, France

http://invs.santepubliquefrance.fr/beh/2018/30-31/pdf/2018_30-31.pdf

 

Psychosocial study on the incentives and obstacles for vaccination in the context of an outbreak of serogroup B invasive meningococcal diseases, Beaujolais (Rhône, France), 2016

Bulletin épidémiologique hebdomadaire, 30-31/2018

9 October 2018, France

http://invs.santepubliquefrance.fr/beh/2018/30-31/pdf/2018_30-31_3.pdf

 

**Respiratory diseases**

 

An international MDR-TB cluster in young refugees from the Horn of Africa

Epidemiologisches Bulletin 48/2018

29 November 2018, Germany

https://www.rki.de/DE/Content/Infekt/EpidBull/Archiv/2018/Ausgaben/48_18.pdf?__blob=publicationFile

Overview of the 2017/18 influenza season in Ireland

Epi-Insight 2018;19(11)

November 2018, Ireland

http://ndsc.newsweaver.ie/epiinsight/t5xi2snufyvn0ykqgxo0gg?email=true&a=2&p=54109352&t=17517804

 

Influenza activity in France, season 2017-2018

Bulletin épidémiologique hebdomadaire, 34/2018

18 October 2018, France

http://invs.santepubliquefrance.fr/beh/2018/34/pdf/2018_34_1.pdf

 

**Sexually transmitted diseases**

Giardiasis in men who have sex with men

Epi-Insight 2018;19(12)

December 2018, Ireland

http://ndsc.newsweaver.ie/epiinsight/9km5dbhfg6w10gkzp9yxn5?a=2&p=54259398&t=17517804

 

Special edition: World AIDS Day, December 1, 2018, “Know your status”

Bulletin épidémiologique hebdomadaire, 40-41/2018

27 November 2018, France

http://invs.santepubliquefrance.fr/beh/2018/40-41/pdf/2018_40-41.pdf

 

Estimating the number of new HIV infections and the total number of people living with HIV in Germany

Epidemiologisches Bulletin 47/2018

22 November 2018, Germany

https://www.rki.de/DE/Content/Infekt/EpidBull/Archiv/2018/Ausgaben/47_18.pdf?__blob=publicationFile

 

Continued increase in syphilis infections in men who have sex with men

Epidemiologisches Bulletin 46/2018

15 November 2018, Germany

https://www.rki.de/DE/Content/Infekt/EpidBull/Archiv/2018/Ausgaben/46_18.pdf?__blob=publicationFile

 

The impact of the worldwide outbreak of hepatitis A in MSM on the epidemiology of hepatitis A in the Netherlands

Infectieziekten Bulletin 2018; 29(8)

October 2018, the Netherlands

https://magazines.rivm.nl/2018/10/infectieziekten-bulletin/het-effect-van-de-wereldwijde-uitbraak-van-hepatitis-onder-msm-op

 

**Zoonoses and vector-borne diseases**

Common animal associated infections quarterly report (England and Wales): third quarter 2018Health Protection Report; 12(41)

16 November 2018, United Kingdom

https://assets.publishing.service.gov.uk/government/uploads/system/uploads/attachment_data/file/757077/hpr4118_zoos.pdf

 

**Other**

Travel-associated diseases 2017

Epidemiologisches Bulletin 44/2018

1 November 2018, Germany

https://www.rki.de/DE/Content/Infekt/EpidBull/Archiv/2018/Ausgaben/44_18.pdf?__blob=publicationFile

 

Twitter: a complementary tool for monitoring the seasonal influenza epidemic in metropolitan France and the regions?

Bulletin épidémiologique hebdomadaire, 34/2018

18 October 2018, France

http://invs.santepubliquefrance.fr/beh/2018/34/pdf/2018_34_2.pdf

